# Influence of height, weight and obesity on risk of breast cancer in an unselected Swedish population.

**DOI:** 10.1038/bjc.1977.263

**Published:** 1977-12

**Authors:** H. O. Adami, A. Rimsten, B. Stenkvist, J. Vegelius

## Abstract

A number of recent studies have shown an association between breast-cancer risk and height, weight and dietary habits, especially fat consumption. In the present study, height and weight were determined for 179 consecutive, unselected, breast-cancer patients and age-matched controls selected from a computerized population register. Height and weight for these two groups were compared, including two different indices for overweight (Quetelet's index and Broca's index). Comparisons were repeated after subdivision into pre- and postmenopausal women. In all calculations, the mean values of patients and controls were very similar and without significant difference. It therefore seems improbable that increased height and weight or obesity constitute risk factors for breast cancer. Earlier studies may have shown differences as the result of selection mechanisms not present in this study.


Br. J. Cancer (1977) 36, 787

### INFLUENCE OF HEIGHT, WEIGHT AND OBESITY ON RISK OF BREAST CANCER IN AN UNSELECTED SWEDISH POPULATION

H. O. ADAMI*, Å. RIMSTEN*, B. STENKVIST† AND J. VEGELIUS‡

*From the *Departments of Surgery and †Clinical Cytology, University Hospital, Uppsala, and the ‡Department of Statistics, Uppsala University, Uppsala, Sweden*

Received 6 June 1977 Accepted 8 August 1977

**Summary.—A number of recent studies have shown an association between breast-cancer risk and height, weight and dietary habits, especially fat consumption. In the present study, height and weight were determined for 179 consecutive, unselected, breast-cancer patients and age-matched controls selected from a computerized population register. Height and weight for these two groups were compared, including two different indices for overweight (Quetelet's index and Broca's index). Comparisons were repeated after subdivision into pre- and postmenopausal women. In all calculations, the mean values of patients and controls were very similar and without significant difference. It therefore seems improbable that increased height and weight or obesity constitute risk factors for breast cancer. Earlier studies may have shown differences as the result of selection mechanisms not present in this study.**

EPIDEMIOLOGICAL investigations have shown great international variations in breast-cancer incidence (Cancer Incidence in Five Continents 1970) but have failed to indicate any factor that would explain these differences satisfactorily. The finding that women migrating from low-risk to high-risk countries gradually approach the risk in the new country (Cancer Incidence, 1970; MacMahon, Cole and Brown, 1973) implicated environmental factors as important. Differences between ethnic groups within the same country, as in South Africa, Israel or Hawaii, supported the hypothesis that cultural characteristics were more important than the total environment (Hill, Goddard and Williams 1971). An association between reproductive life and breast-cancer risk has been shown in many studies. The association was, however, not strong enough to explain the international differences (Wynder, Bross and Hirayama, 1960; MacMahon *et al.*, 1973) and has been questioned in a recent study (Adami *et al.*, 1977).

The hypothesis that breast-cancer risk is associated with dietary factors has attracted interest (Wynder, 1968). It has been supported by several authors who compared different countries and found a significant correlation between fat consumption and breast-cancer incidence as well as breast-cancer mortality (Lea, 1966; Carrol, Gammal and Plunkett, 1968; Wynder, 1968; Drasar and Irving, 1973; Armstrong and Doll, 1975). It has also, however, been shown by Armstrong and Doll (1975) that breast-cancer incidence was better correlated to GNP (Gross national product) ($r = 0{\cdot}83$) than to total fat consumption ($r = 0{\cdot}79$) or any other dietary factor. This underlines the fact that the question of a causal relationship has to be considered with care. However, such a relationship has been supported by experimental research in the rat, where the yield of mammary tumours increased with

Address for reprints: Hans-Olov Adami, MD., Department of Surgery, University Hospital, S-700 14 Uppsala, Sweden fat level in the diet (Carrol *et al.*, 1968). A higher frequency of overweight women was also observed among breast-cancer patients than among controls in a number of studies (De Waard, Baanders-Van Halewijn and Huizinga, 1964; Valaoras *et al.*, 1969; Mirra, Cole and MacMahon, 1971; De Waard and Baanders-Van Halewijn, 1974; Basu and Williams, 1975).

The result of the study described here is, however, in contradiction to those studies indicating obesity as a risk factor for cancer.

### PATIENTS, CONTROLS AND METHODS

*Patient group.*—The patient group consisted of 179 patients with breast cancer diagnosed consecutively from October 1975–March 1976 in 4 Swedish counties. The population in this area is uniform in race and nationality. The mean age was 63 and the median age 64 years. The patients were staged according to the TNM classification (UICC, 1974) (Table I).

TABLE. I.—*Classification of Patients According to the TNM System (UICC* 1974)

| Stage | Number of women | % |
|---|---|---|
| I | 70 | 39 |
| II | 85 | 47 |
| III | 16 | 9 |
| IV | 8 | 5 |
| | 179 | 100 |

The study was organized in cooperation with 10 surgical and 2 oncological clinics, in addition to the University Hospital in Uppsala. All diagnosis and therapy of breast cancer within the studied area took place at these clinics. Height and weight were obtained from a questionnaire answered by all patients in connection with admission to the hospital Only 2 of the breast-cancer patients diagnosed during the observation period refused participation, (*i.e.* 2/181 or $\sim 1\%$).

*Control group.*—The control group consisted of 179 women matched by age to the breast-cancer group. In Sweden there are unique possibilities for selecting age-matched controls from a computerized population register. To be able to replace expected drop-outs with new controls, the 4 women in each county closest in age to the respective breast cancer patient were selected. These were randomly labelled as "control alternative" 1, 2, 3 and 4, respectively. The alternatives 2, 3 or 4 were included in the study only when the previous one had a history of breast cancer or refused participation in the study.

A letter was sent to the controls, in which they were informed of the investigation and asked to answer an attached questionnaire. This included questions concerning height and weight in addition to a large number of epidemiological factors, and was identical with the questionnaire given to the patients.

All women included in the control group were personally examined by one of the investigators (HOA) at the office of the district nurse. The intention was, among other things, to accurately measure height and weight, to draw blood samples for hormone analysis and to verify and complete information in the questionnaire by personal interview.

In 154 instances, the first alternative was included in the control group and the primary loss was thus 25 women (14%). These losses were replaced by alternative 2 in 21 instances and by alternative 3 in 4. As a result, each breast-cancer patient received one age-matched control.

A bias introduced by the 25 lost primary controls could not be definitely excluded, but was shown to be improbable with the help of two types of estimation, presented more extensively in a special study (Adami and Vegelius, 1977). First, there were no significant differences between the lost controls and those who replaced them, with respect to those factors where information could be obtained from the computerized population register (*i.e.* marital status and place of residence). Second, the influence of the lost controls was estimated with the help of those 43 persons in the definite control group who were reluctant and accepted participation only after persuasion. These women would have been included in the lost group without a special effort including repeated letters and telephone calls. This estimation did not indicate a significant difference with respect to height and weight between the unwilling controls and those who immediately accepted participation.

*Pre- and postmenopause.*—In subdividing the total groups into pre- and postmenopausal women, the history given by them was in all instances verified by determination of the serum level of follicle-stimulating hormone (FSH). Values above 3·0 $\mu$g/l were considered conclusive of menopause (Wide *et al.*, 1973). Thirty patients and 28 controls were premenopausal.

*Weight indices.*—In order to measure obesity independent of height, two weight indices were used:

(1) $W_Q = C\frac{W}{H^2}$ (Quetelets's index) (Khosla and Lowe, 1967) where W is the weight in kg and H is the height in cm and C is a constant, arbitrarily chosen as $C = 10^2$

(2) $W_B = C\frac{W}{0{\cdot}9\,(H-100)}$ (Broca's index) (Werner 1977, personal communication) with the same definitions as above.

The criterion of no correlation between index and height, discussed by Khosla and Lowe (1967) and others, was better fulfilled by Quetelet's index ($r = 0{\cdot}022$) than by Broca's index ($r = -0{\cdot}190$).

### STATISTICAL METHODS

The differences concerning height, weight and weight indices between the patient and the control groups were tested with the *t* test for paired samples. This is the uniformly most applicable test when it is used one-tailed (Brownlee, 1965). Variables who did not fulfil the normal distribution assumption were tested with the nonparametric Wilcoxon's test for related samples (Siegel, 1956). The product-moment correlation coefficients were calculated, together with the two-tailed *P*-values of the independence hypothesis (Kendall and Stuart, 1961). A *P*-value of 0·05 was generally accepted as the level of significance.

### RESULTS

There were no differences in the distribution of height and weight between the patient and control groups (Figs. 1 and 2). Detailed results of the statistical

FIG. 1.—Distribution of height in 177 patients and 179 controls.

FIG. 2.—Distribution of weight in 178 patients and 179 controls.

calculations concerning height, weight and the 2 weight indices are shown in Table II for the whole groups and after subdivision into pre- and postmenopausal women. The number of pairs where information was lacking for one or both in the pair is shown for each variable. The mean values were very close to each other and the differences far from significant. They remained insignificant even if a one-tailed *t* test was used.

Factors that could obscure a real difference between the groups were con-

TABLE II.—*Comparison between Patient and Control Group with Respect to Height, Weight, Quetelet's Index and Broca's Index*

| Variable | Meno-pausal status | No of pairs in calculation / No of pairs in group | Mean P* | Mean C† | s.d. P* | s.d. C† | P (t-test) |
|---|---|---|---|---|---|---|---|
| Height | total | 176/179 | 161·73 | 161·36 | 5·58 | 6·49 | 0·56 |
| (cm) | pre | 30/30 | 164·13 | 164·43 | 5·30 | 5·40 | 0·84 |
| | post | 145/149 | 161·24 | 160·73 | 5·53 | 6·54 | 0·48 |
| Weight | total | 177/179 | 65·55 | 65·75 | 10·9 | 11·7 | 0·87 |
| (kg) | pre | 30/30 | 62·13 | 64·70 | 8·17 | 11·46 | 0·28 |
| | post | 147/149 | 66·25 | 65·96 | 11·27 | 11·77 | 0·83 |
| Quetelet's | total | 175/179 | 0·251 | 0·252 | 0·041 | 0·040 | 0·89 |
| index | pre | 30/30 | 0·230 | 0·238 | 0·026 | 0·033 | 0·29 |
| | post | 145/149 | 0·255 | 0·254 | 0·043 | 0·041 | 0·83 |
| Broca's | total | 175/179 | 118·6 | 119·1 | 20·1 | 19·1 | 0·81 |
| index | pre | 30/30 | 107·7 | 111·3 | 12·1 | 14·8 | 0·32 |
| | post | 145/149 | 120·9 | 120·7 | 20·7 | 19·5 | 0·95 |

* Patient group

† Control group sidered. First, the women with advanced breast cancer (clinical stage IV) might have a reduced weight due to their disease. A separate calculation, with these women and their controls excluded, did not reveal any significant difference. Second, the correlation coefficient was calculated between the factors shown in Table II and a large number of factors relating to socio-economic circumstances and reproductive life. There was a slight but significant negative correlation between weight and education ($r = -0{\cdot}150$, $P = 0{\cdot}045$). The breast-cancer patients also had, on average, a longer education than the controls (Wilcoxon's test, $P = 0{\cdot}04$). This might conceal a real tendency to overweight in the patient group. Such an influence could, however, be excluded when the calculation was repeated using only those 134 pairs without differences in education between patient and control.

### DISCUSSION

Sweden is a high-risk country for breast cancer. A correlation between increased height, weight and of obesity and breast cancer incidence has been shown in different countries with great variation in incidence. This makes it probable that these factors would be relevant in Sweden as well. The inability of this study to confirm earlier results is therefore surprising. In discussing its causes we have to consider the characteristics of the patient group as well as the control group.

To avoid bias due to selective factors among the patients, an unselected material is necessary. The Swedish medical system, with all in-patients treated by a public medical service in one hospital within each area, offers the prerequisites to collect such material. The loss in the patient group does not exceed the 2 cases already accounted for.

The most important difference, however, between this study and most of the previous ones is the characteristics of the control group. The Swedish computerized population register contains information about all individuals living within the respective areas. This gave us an excellent opportunity to choose our controls from the whole female population in each county. It also made it possible to achieve exact age-matching, which is important, considering the changes in weight during life. Thus, each breast cancer patient had an exactly age-matched control. The patient group, as well as the control group, constituted a homogeneous, Caucasian population which was unselected with respect to marital status, socio-economic status, place of residence, parity, age or stage of disease.

The 25 primary controls lost were considered as a possible source of error.

Our calculations, presented in a special study (Adami and Vegelius, 1977) showed this to be improbable. Inaccuracies in the answers given could not be definitely excluded. Observations made in the control group in connection with the examination did, however, indicate that the differences between measured weight and height and answers given in the questionnaire were very small. The information calculated upon was therefore considered to be reliable, and possible sources of bias in previous studies have to be considered.

A number of studies have indicated increased weight and height (Valaoras *et al.*, 1969; Mirra *et al.*, 1971; Lin, Chen and MacMahon, 1971; Ravinihar, MacMahon and Lindtner, 1971; Basu and Williams, 1975) as well as obesity reflected in high values of the indices weight/height (Basu and Williams 1975) and weight/height$^2$ (Valaoras *et al.*, 1969; Mirra *et al.*, 1971) as risk factors for breast cancer. In these investigations, the heaviest and tallest women had about a two-fold increase in breast-cancer risk. Others, however, did not show any difference in weight (Wynder 1968) or Quetelet's index (Stavraky and Emmons, 1974) between patients and controls.

In all the above studies, the controls were selected from hospital patients, a practice which can be hazardous in epidemiological research. The degree of bias introduced by hospital patients as controls is impossible to estimate, especially if the distribution of diagnoses is unknown, as in the recent international collaborative study (Valaoras *et al.*, 1969; Mirra *et al.*, 1971; Ravnihar *et al.*, 1971). This bias is probably considerable if a number (Wynder *et al.*, 1960; Lin *et al.*, 1971) or even a majority (Stavraky and Emmons, 1974; Basu and Williams, 1975) of the controls have a malignant or other type of disease. As far as height is concerned, appreciable differences exist between social classes (Khosla and Lowe, 1967). Those in a higher socio-economic class have on average a greater height than those in a lower. Persons in the lower classes also have a higher morbidity from a large number of diseases (Syme and Berkman, 1976). This could explain a lower mean height in hospital patients than in the general population.

In the studies of De Waard (De Waard *et al.*, 1964; De Waard and Baanders-van Halewijn, 1974) increased height and weight were considered as risk factors. A combination of height over 165 cm and weight over 70 kg resulted in a three- to four-fold increase in breast cancer risk (De Waard and Baanders-van Halewijn, 1974). In these studies, hospital patients were not utilized as controls, and the reason for the disagreement between their results and those in the present study is therefore less obvious. Theoretically it could be explained if the Dutch population is heterogeneous, containing ethnic subgroups who differ with respect to breast-cancer incidence as well as height and weight, without these factors having a causal relationship. It could also be argued that the relative risks were calculated on very small differences. This might be the reason why the calculation of Quetelet's index did not reveal any difference between patients and controls despite this index being highly correlated with weight (Khosla and Lowe, 1967).

In summary, the present investigation contradicts the assumption that increased height and weight or obesity constitute risk factors for breast cancer.

This study was supported by contract NO1-CB1 53968 from the National Cancer Institute, U.S.A.

### REFERENCES

Adami, H.-O., Rimsten, Å., Stenkvist, B. & Vegelius, J. (1977) Reproductive History and Risk of Breast Cancer: A Case-control Study in an Unselected Swedish Population. *Cancer, N.Y.* (in press).

Adami, H. O. & Vegelius, J. (1977) A Method for Estimating Bias Introduced into Clinical Investigations by those who Refuse Participation. *Ann. clin. Res.* (submitted).

Armstrong, B. & Doll, R. (1975) Environmental Factors and Cancer Incidence and Mortality in

Different Countries: with Special Reference to Dietary Practices. *Int. J. Cancer*, **15**, 617.

BASU, T. K. & WILLIAMS, D. C. (1975) Plasma and Body Lipids in Patients with Carcinoma of the Breast. *Oncology*, **31**, 172.

BROWNLEE, K. A. (1965) *Statistical Theory and Methodology in Science and Engineering.* 2nd ed. New York: John Wiley.

CANCER INCIDENCE IN FIVE CONTINENTS (1970) Vol. 2. Eds. R. Doll, C. Muir and J. Waterhouse. Berlin–Heidelberg–New York: Springer-Verlag.

CARROL, K. K., GAMMAL, E. B. & PLUNKETT, E. R. (1968) Dietary Fat and Mammary Cancer. *Can. med. Ass. J.*, **98**, 590.

DE WAARD, F., BAANDERS-VAN HALEWIJN, E. A. & HUIZINGA, J. (1964) The Bimodal Age Distribution of Patients with Mammary Carcinoma. *Cancer, N.Y.*, **17**, 141.

DE WAARD, F. & BAANDERS-VAN HALEWIJN, E. A. (1974) A Prospective Study in General Practice on Breast Cancer Risk in Postmenopausal Women. *Int. J. Cancer*, **14**, 153.

DRASAR, B. S. & IRVING, I. (1973) Environmental Factors and Cancer of the Colon and Breast. *Br. J. Cancer*, **27**, 167.

HILL, M. J., GODDARD, P. & WILLIAMS, R. E. O. (1971) Gut Bacteria and Aetiology of Cancer of the Breast. *Lancet*, ii, 472.

International Union Against Cancer (IUCC) (1974) *TNM Classification of malignant tumours.* 2nd ed. Geneva.

KENDALL, N. G. & STUART, A. (1961) *The Advanced Theory of Statististics.* Vol. 2, London: Charles Griffin. p. 296.

KHOSLA, T. & LOWE, C. R. (1967) Indices of Obesity Derived from Body Weight and Height. *Br. J. prev. soc. Med.*, **21**, 122.

LEA, A. J. (1966) Dietary Factors Associated with Death-rates from certain Neoplasms in Man. *Lancet*, ii, 332.

LIN, T. M., CHEN, K. P. & MACMAHON, B. (1971) Epidemiologic Characteristics of Cancer of the Breast in Taiwan. *Cancer, N.Y.*, **27**, 1497.

MACMAHON, B., COLE, P. & BROWN, J. (1973) Etiology of Human Breast Cancer: A Review. *J. natn. Cancer Inst.*, **50**, 21.

MIRRA, A. P., COLE, P. & MACMAHON, B. (1971) Breast Cancer in an Area of High Parity: Sao Paulo, Brazil. *Cancer, Res.* 31, 77.

RAVNIHAR, B., MACMAHON, B. & LINDTNER, J. (1971) Epidemiologic Features of Breast Cancer in Slovenia. 1965–1967. *Eur. J. Cancer*, **7**, 295.

SIEGEL, S. (1956) *Nonparametric Statistics for the Behavioural Sciences.* Tokyo: McGraw-Kogakusha Ltd. p. 75.

STAVRAKY, K. & EMMONS, S. (1974) Breast Cancer in Premenopausal and Postmenopausal Women. *J. natn. Cancer Inst.*, **53**, 647.

SYME, S. L. & BERKMAN, L. F. (1976) Social Class, Suspectibility and Sickness. *Am. J. Epidemiol.*, **104**, 1.

VALAORAS, V. G., MACMAHON, B., TRICHOPOULOS, D. & POLYCHRONOMPOULOU, A. (1969) Lactation and Reproductive Histories of Breast Cancer Patients in Greater Athens, 1965–67. *Int. J. Cancer* **4**, 350.

WIDE, L., NILLIUS, S. J., GEMZELL, C. & ROOS, P. (1973) Radioimmunosorbent Assay of Follicle-stimulating Hormone and Luteinizing Hormone in Serum and Urine from Men and Women. *Acta Endocrinol.*, Suppl. 174. p. 1.

WYNDER, E. L. (1968) Current Concepts of the Aetiology of Breast Cancer. In *Prognostic Factors in Breast Cancer.* Eds A. P. M. Forrest and P. B. Kunkler. Edinburgh and London: E. S. Livingstone Ltd, p. 32.

WYNDER, E., BROSS, I. J. & HIRAYAMA, T. (1960) A Study of the Epidemiology of Cancer of the Breast. *Cancer, N.Y.*, **13**, 559.


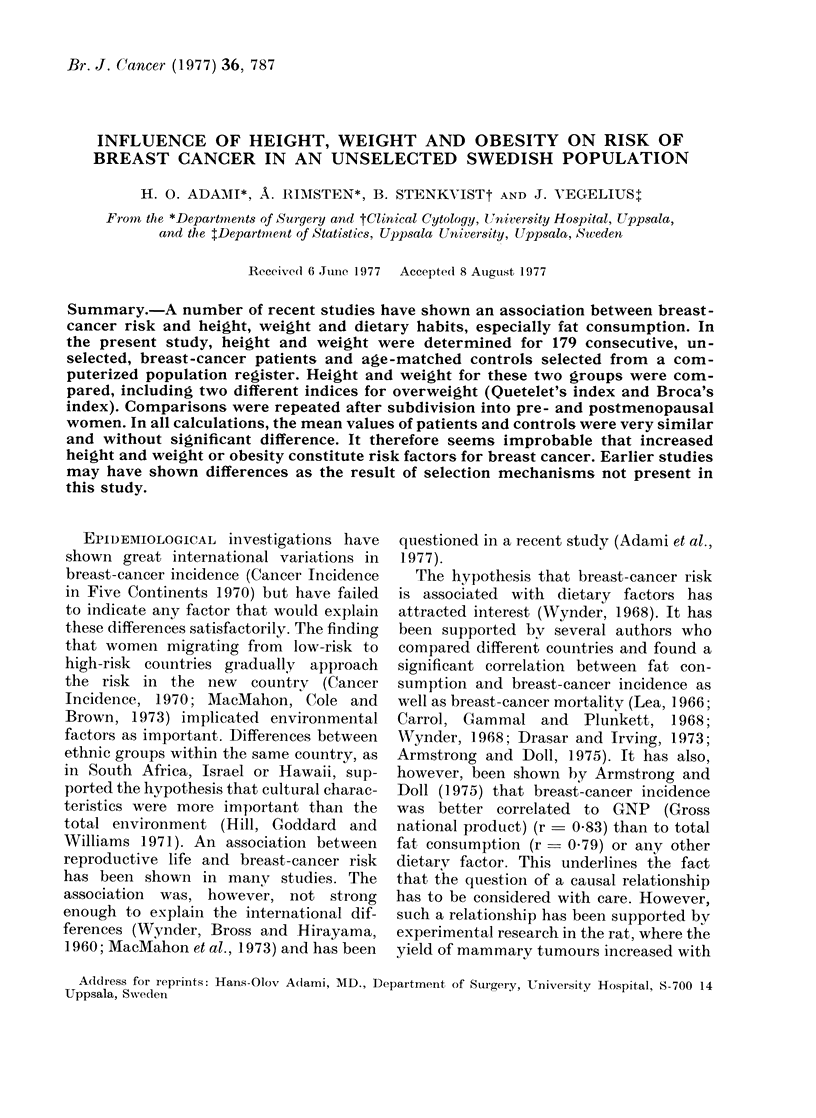

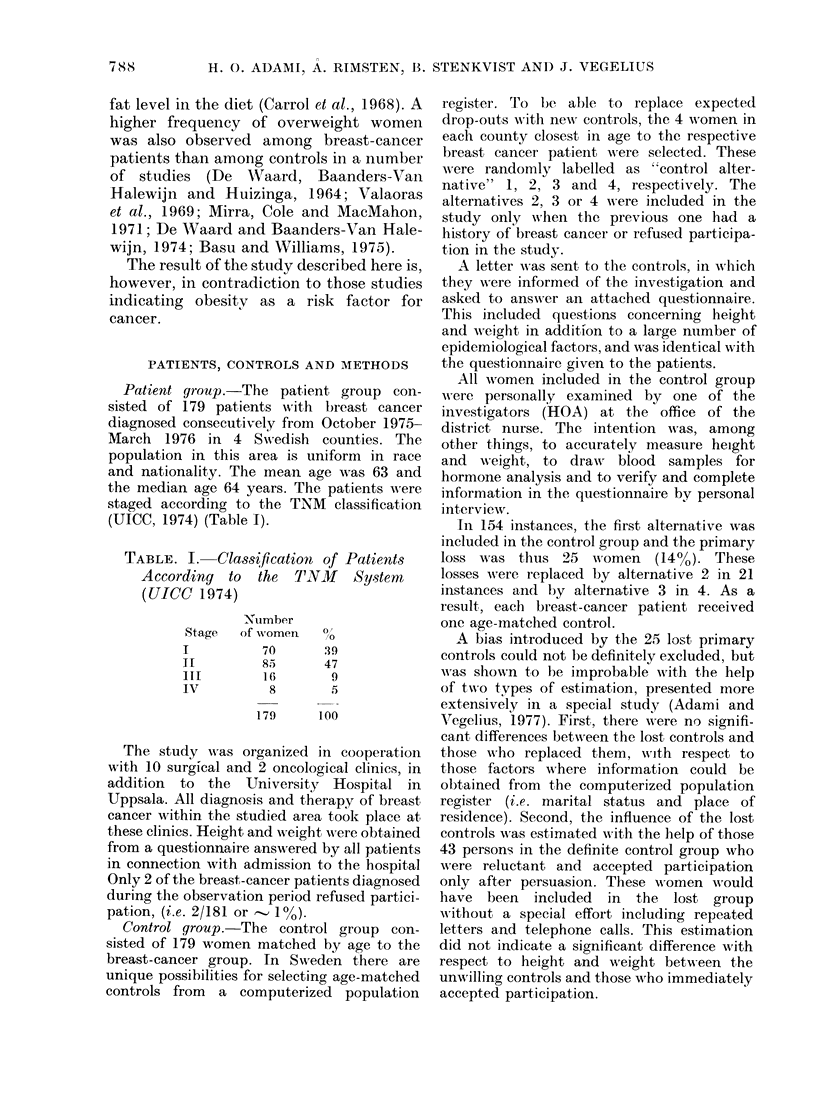

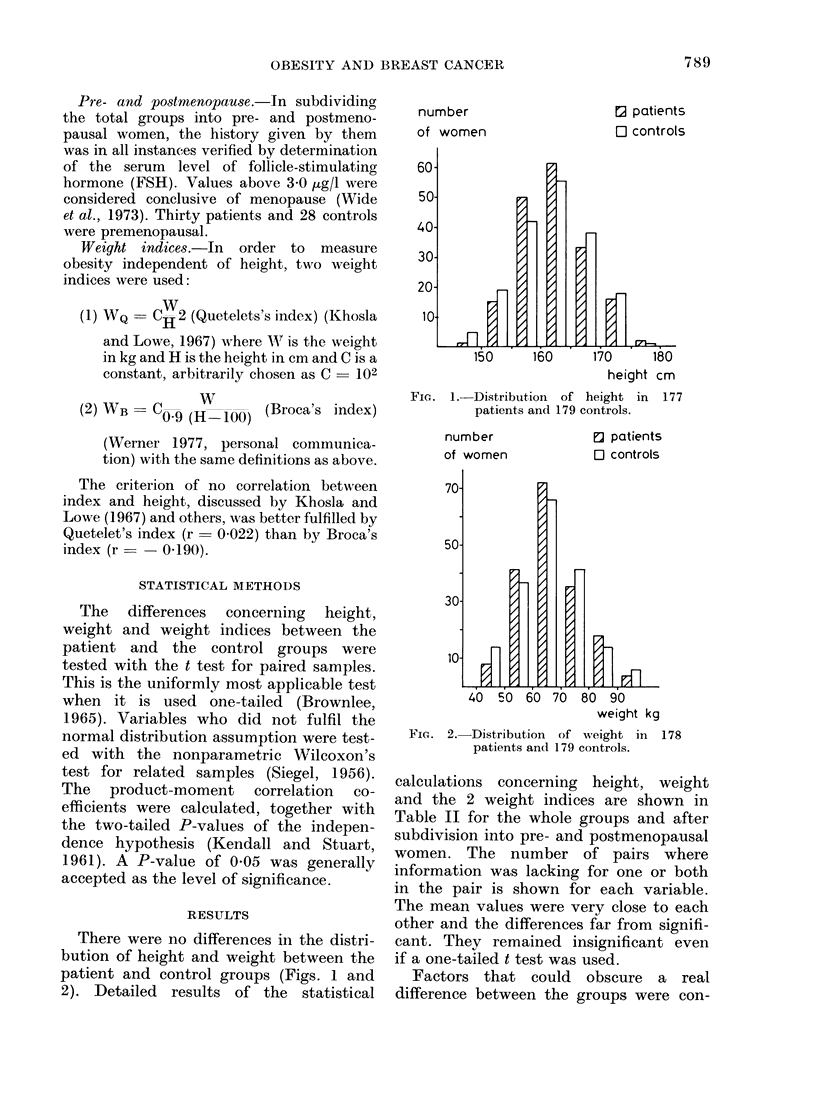

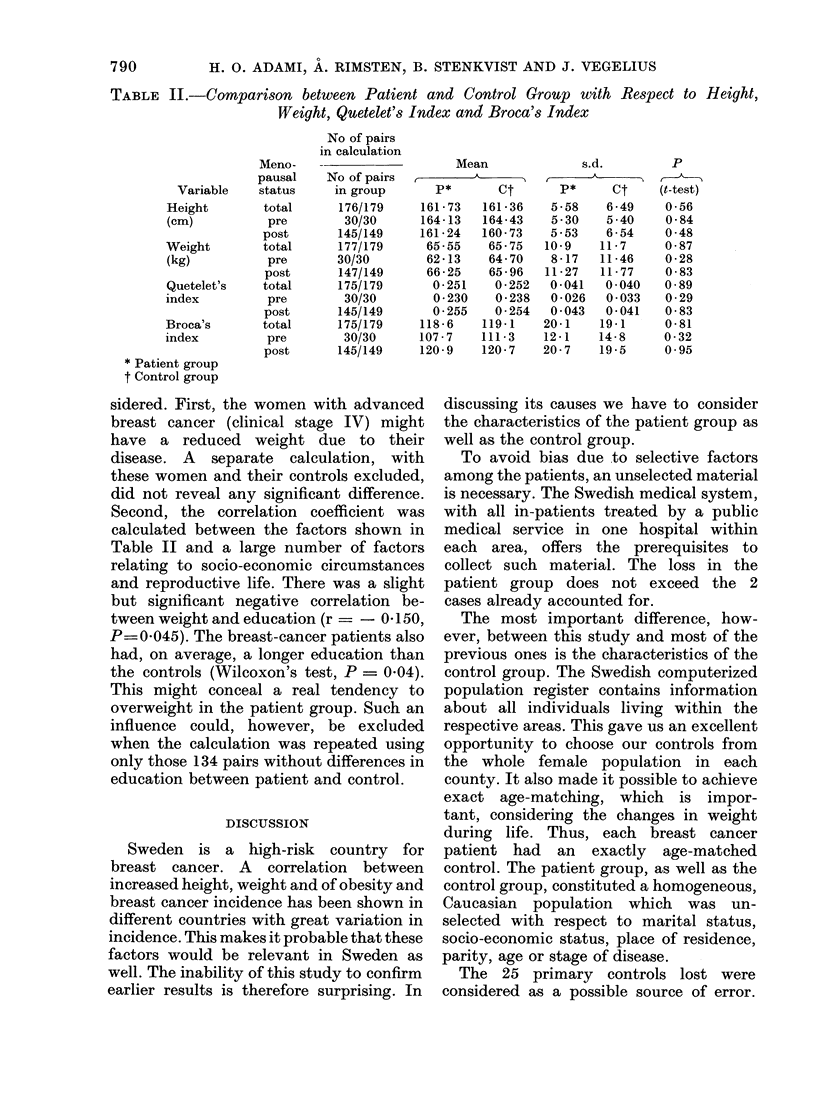

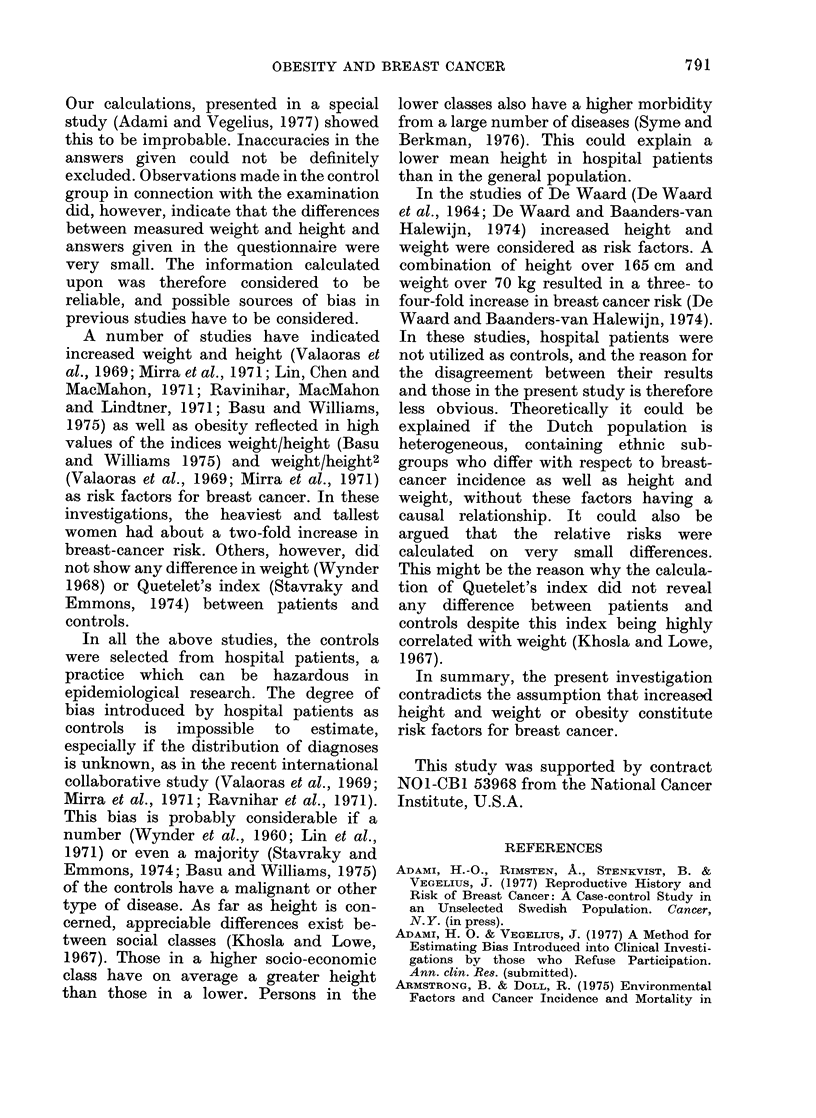

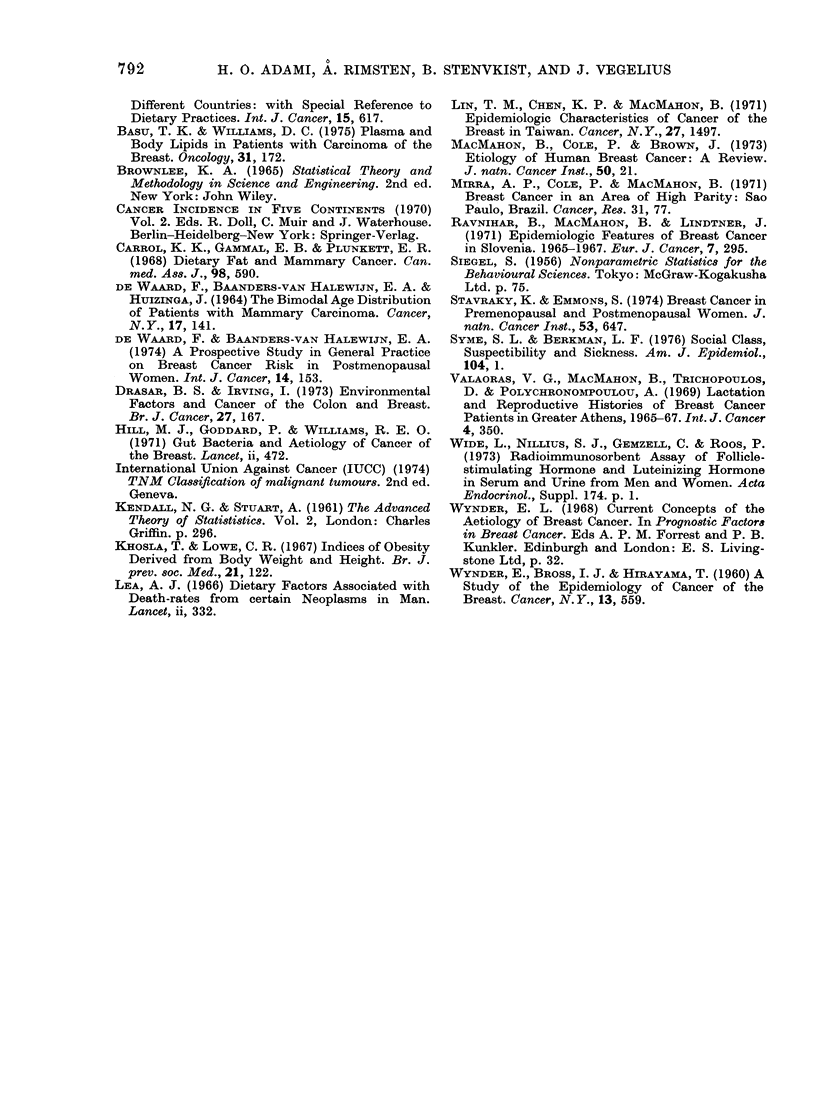


## References

[OCR_00672] Armstrong B., Doll R. (1975). Environmental factors and cancer incidence and mortality in different countries, with special reference to dietary practices.. Int J Cancer.

[OCR_00681] Basu T. K., Williams D. C. (1975). Plasma and body lipids in patients with carcinoma of the breast.. Oncology.

[OCR_00696] Carroll K. K., Gammal E. B., Plunkett E. R. (1968). Dietary fat and mammary cancer.. Can Med Assoc J.

[OCR_00701] DE WAARD F., BAANDERS-VANHALEWIJN E. A., HUIZINGA J. (1964). THE BIMODAL AGE DISTRIBUTION OF PATIENTS WITH MAMMARY CARCINOMA; EVIDENCE FOR THE EXISTENCE OF 2 TYPES OF HUMAN BREAST CANCER.. Cancer.

[OCR_00713] Drasar B. S., Irving D. (1973). Environmental factors and cancer of the colon and breast.. Br J Cancer.

[OCR_00718] Hill M. J., Goddard P., Williams R. E. (1971). Gut bacteria and aetiology of cancer of the breast.. Lancet.

[OCR_00733] Khosla T., Lowe C. R. (1967). Indices of obesity derived from body weight and height.. Br J Prev Soc Med.

[OCR_00738] Lea A. J. (1966). Dietary factors associated with death-rates from certain neoplasms in man.. Lancet.

[OCR_00743] Lin T. M., Chen K. P., MacMahon B. (1971). Epidemiologic characteristics of cancer of the breast in Taiwan.. Cancer.

[OCR_00758] Ravnihar B., MacMahon B., Lindtner J. (1971). Epidemiologic features of breast cancer in Slovenia, 1965-1967.. Eur J Cancer.

[OCR_00768] Stavraky K., Emmons S. (1974). Breast cancer in premenopausal and postmenopausal women.. J Natl Cancer Inst.

[OCR_00773] Syme S. L., Berkman L. F. (1976). Social class, susceptibility and sickness.. Am J Epidemiol.

[OCR_00778] Valaoras V. G., MacMahon B., Trichopoulos D., Polychronopoulou A. (1969). Lactation and reproductive histories of breast cancer patients in greater Athens, 1965-67.. Int J Cancer.

[OCR_00799] WYNDER E. L., BROSS I. J., HIRAYAMA T. (1960). A study of the epidemiology of cancer of the breast.. Cancer.

[OCR_00707] de Waard F., Baanders-van Halewijn E. A. (1974). A prospective study in general practice on breast-cancer risk in postmenopausal women.. Int J Cancer.

